# Language impairments in behavioural variant frontotemporal dementia: a comparative study with semantic dementia

**DOI:** 10.1007/s00415-026-14064-w

**Published:** 2026-08-22

**Authors:** Sharon Savage, Ohnmar Aung, David Foxe, Olivier Piguet

**Affiliations:** 1 School of Sciences, Discipline of Psychological Sciences, The University of Newcastle, Callaghan, NSW 2308 Australia; 2 School of Psychology; Brain and Mind Centre, The University of Sydney, Sydney, NSW 2050 Australia

**Keywords:** Frontotemporal dementia, Language, Semantic dementia, Single-word processing, Naming

## Abstract

**Introduction:**

Recent evidence suggests that individuals with behavioural variant frontotemporal dementia (bvFTD) may experience language changes in a similar, but less severe manner, than what is observed in semantic dementia (SD). Few studies, however, have specifically compared these patient groups or considered the cognitive underpinnings. To explore this in more depth, this study aimed to profile patterns of language performance within both "probable" and "possible" categories of bvFTD and to compare them to SD, while also considering the relationships between language and executive function measures.

**Methods:**

Participants included 106 probable bvFTD, 43 possible bvFTD, 42 SD, and 112 healthy controls from the FRONTIER clinic. Data analysed included the Addenbrooke’s Cognitive Examination (ACE-III), single word processing (SYDBAT) and executive function measures (word generativity, mental flexibility, response inhibition and working memory).

**Results:**

Naming and semantic association impairments frequently occurred in probable bvFTD (51% and 49% respectively). Approximately one quarter of possible bvFTD patients also showed language impairments. The SD pattern of pronounced naming impairment as compared to other single word tasks, was not found in the bvFTD groups. Semantic measures from the ACE-III predicted language performance in bvFTD groups; response initiation and suppression also emerged as a significant predictor.

**Conclusion:**

Naming and semantic association deficits commonly arise in probable bvFTD, but may also be observed in some patients with possible bvFTD. The pattern of impairments in bvFTD were not wholly consistent with those seen in SD. Both semantic and executive function measures may be associated with language performance deficits observed in bvFTD.

## Highlights

- Naming impairments commonly occur in behavioural variant FTD
- Semantic association deficits also occur in probable FTD
- Semantic memory and executive function predict language performance in bvFTD

## Introduction

Frontotemporal dementia (FTD) is a progressive brain condition defined by three canonical clinical presentations. These include a behavioural variant (bvFTD) [1], as well as two forms of primary progressive aphasia (PPA): semantic dementia (SD; or the semantic variant of PPA), and progressive nonfluent aphasia (or the non-fluent/agrammatic variant of PPA) [2]. While language features co-occur with behavioural symptoms [3], language changes in bvFTD have received limited attention to-date. This under-appreciation of language change may, in part, be due to the diagnostic criteria for bvFTD focusing on behavioural and functional impairments [1]. Some language change may be expected in this disease, however, given the location of predominant brain atrophy [4] and known associations between language performance and both temporal lobe atrophy [5–7] and frontotemporal activity [8].

Investigating language integrity in bvFTD is important for a number of reasons. Firstly, deficits may emerge early in the disease and affect daily functioning [9, 10]. Secondly, assessment of language in bvFTD may prove a helpful aid to clinical diagnosis. Reduced picture naming has been found in bvFTD patients with abnormal MRIs (i.e., “probable bvFTD”) but not in “MRI-normal” bvFTD patients (i.e., “possible bvFTD”) [11]. In cases where brain imaging is not available or possible, this clinical marker could help provide further certainty regarding the diagnostic category. In addition, bvFTD is known to arise from a number of pathologies that are difficult to distinguish in life [12, 13]. Some people with possible bvFTD may indeed have an alternative psychiatric condition, which becomes evident over time. Recent studies suggest that the examination of both expressive and receptive language may aid in the differentiation of bvFTD from other psychiatric conditions at presentation [14]. These findings suggest that examination of language integrity in bvFTD could help with diagnostic decisions.

To enable the development of language markers to guide diagnosis, separate subgroup analyses are needed when investigating language skills in bvFTD. To-date, few studies have clearly included both possible and probable bvFTD groups, with most either not mentioning the distinction or focusing solely on probable cases (see review by Geraudie et al. [15]). Studies that have analysed both possible and probable cases (or equivalents, e.g., “MRI-normal” vs “MRI-abnormal”; “bvFTD/PET+” vs “bvFTD/PET– “) have, to-date, reported conflicting findings regarding language deficits [11, 16, 17], signalling the need for further investigation.

### Single-word processing in frontotemporal dementia

Assessment of language at the single-word level has been used effectively to characterise and distinguish impairments within language-led dementias (i.e., PPA) [18, 19]. In bvFTD, single-word-level language impairments have been identified in naming [15, 20] (found in 31 of 49 studies reviewed [15]), word comprehension (8 of 12 studies), and semantic knowledge (6 of 8 studies), but not word repetition (0 of 5 studies [15]). In two studies that compared bvFTD with other FTD subtypes, bvFTD patients showed less severe but similar deficits to individuals with SD [5, 6] (i.e., reductions in naming, word comprehension, and semantic association, but relative sparing of word repetition). Whether the inter-relationships between the naming and single-word comprehension deficits were similar between the groups, or underpinned by similar mechanisms, was not examined. Also, in many studies, small sample sizes (e.g., n < 30 participants) have impeded the ability to conduct more complex investigations within datasets.

In SD, deficits in naming and word comprehension are known to arise from degradation of the semantic memory system, associated with significant atrophy to the anterior and medial temporal lobes [2]. For bvFTD, explanations for language changes remain largely unconfirmed. Prior studies have shown, however, that the deficits in word retrieval (naming) and comprehension are not simply explained by general cognitive decline, given deficits remain after accounting for disease severity and non-verbal intellectual skills [5]. In addition, deficits in naming are not only observed late in the disease, but have been reported in pre-symptomatic and early phases [21, 22].

The relative contributions of executive function and linguistic factors to language change are currently undetermined. From an executive function perspective, poor performance could potentially arise from deficits in inhibition, working memory, or cognitive flexibility, all of which are known to occur in bvFTD [23–26]. Disinhibition could lead to more off-task responses, or impulsive errors. Poor working memory could impact lexical processing/language comprehension performance by reducing the ability to retain task requirements in mind [27]. Other forms of executive dysfunction could lead to reduced efficiency in searching the lexicon or increased perseverative responses. In support of an executive contribution, individuals with bvFTD who experience greater language deficits reportedly experience greater executive functioning impairment [5].

Alternatively, language changes in bvFTD may have a primarily linguistic basis and arise in response to the atrophy within key language processing areas of the brain (e.g., lateral temporal, insula, superior temporal, and mid-hippocampal regions) [6, 13]. Further studies that include both language and executive measures in bvFTD are needed to address this question.

### Aims and hypotheses

The current study aimed to examine language abilities in large samples of individuals diagnosed with either possible and probable bvFTD, using a comprehensive assessment of single word language in combination with measures of executive function. In doing so, this study sought to compare language profiles of bvFTD and SD, and investigate the relative contributions of semantic functioning and executive measures to language performance. It was predicted that a greater range and severity of impairment would arise in probable bvFTD as compared to possible bvFTD, and that a broadly similar pattern of performance across single word processing skills would be observed between SD and bvFTD although not necessarily underpinned by the same cognitive abilities. Whereas performance in SD was expected to be primarily associated with semantic impairment, performance in bvFTD was expected to reflect contributions from both semantic and executive functioning. Accordingly, differences between bvFTD and SD were anticipated when examining specific relationships between tasks where different demands on semantic knowledge or executive control may be present.

## Materials and methods

### Participants

Secondary data analysis was conducted on participant data from the research clinic at FRONTIER, the frontotemporal dementia clinical research group at the Brain and Mind Centre of the University of Sydney. Data from all eligible patients presenting between August 2008 and June 2021 were included in the study, resulting in 106 probable bvFTD, 43 possible bvFTD, and 42 SD patients. In addition, 112 healthy controls were included, sourced from FRONTIER’s research volunteer pool. Clinical diagnosis was established by consensus following a comprehensive examination involving clinical interview by an experienced neurologist, a cognitive assessment, and review of neuroimaging, in line with diagnostic criteria for bvFTD [1] and SD [2]. Specifically, to distinguish between possible and probable bvFTD, neuroimaging evidence from 3 T MRI structural brain imaging was closely examined. For all participants classified as possible bvFTD, there was no frontal and/or anterior temporal atrophy sufficient to provide neuroimaging support for a probable diagnosis. In addition, for the 24 possible bvFTD participants who subsequently returned for at least one follow up visit, additional neuroimaging continued to support a diagnosis of possible bvFTD (i.e. no participants converted from possible to probable bvFTD within this sample). Information from previous neuropsychological assessments or clinical imaging provided by the referring clinician was also considered within the diagnostic evaluation. While not used as a primary indicator of SD, we acknowledge that where no previous cognitive testing was available, data from the Sydney Language Battery (see measures below) was part of the information available to the neurologist who confirmed the diagnosis. Healthy controls underwent the same neuropsychological assessments and neuroimaging procedures.

Exclusion criteria included the presence of any additional suspected psychiatric, neurodegenerative or neurological disorder (e.g., bipolar disorder, motor neuron disease), very severe disease stage or limited general cognitive ability: <40/100 on the Addenbrooke’s Cognitive Examination-III [ACE-III][28]; *n* = 7), no formal years of education in English or an inability to provide answers in English (*n* = 9), substantial missing data (> 50% of the language or neuropsychological variables; *n* = 13), and for healthy controls, a score below 88/100 on the ACE-III [29]. For SD patients, an additional exclusion criterion was applied for those participants whose neuroimaging indicated right-sided asymmetric anterior temporal lobe atrophy. This was due to both insufficient numbers to analyse this subgroup separately, as well as lack of diagnostic clarity around this clinical phenotype (also known as right temporal variant of FTD [30]).

All participants or their person responsible (e.g., spouse, family member etc.) provided written informed consent in accordance with the Declaration of Helsinki. Original data collection was approved by the South Sydney Area Health Service, the University of New South Wales and the University of Sydney Human Research Ethics Committees (HREC 2020/224), with this retrospective study approved by University of Newcastle Human Research Ethics Committee (H-2021-0399).

### Assessment measures

Disease severity was evaluated using the informant-based Frontotemporal Dementia Rating Scale (FRS [31]; a measure of functional ability and behaviour). General cognitive ability was assessed using the ACE-III [29] or, if the assessment pre-dated this version, the ACE-Revised [32], with scores converted to ACE-III using a validated algorithm [28]).

To assess language, the Sydney Language Battery (SYDBAT [19]) was used to test picture naming, word comprehension (word-picture matching), semantic association (picture-picture matching), and word repetition. Each subtest is based on the same 30 polysyllabic target words, generating a subtest total score of 30, where higher scores denote better performance. For the semantic association task, this involves matching a picture of each target word with a picture of an associated item. Across the four tasks, each item was scored as either correct or incorrect. The SYDBAT has been validated in dementia populations, with high convergent validity and reliability [18, 19]. In addition, naming and comprehension items of the ACE-III were combined to form a “ACE-III Semantic” measure out of 16.

Participants also completed neuropsychological tests of executive function. The available measures from the existing dataset included: Digit Span subtest of the Wechsler Adult Intelligence Scale – Third edition (WAIS-III [33]); Trail Making Test – Part A and B (TMT [34]); plus two verbally mediated tasks—Hayling Sentence Completion Test [35], and letter fluency using the letters ‘F’, ‘A’ and ‘S’ [36]. From these tests, four key executive functioning measures were extracted. While we acknowledge that no test provides a pure measure of executive function, the maximum span achieved from the Digit Span backwards subtest (DSB) was calculated as an indicator of working memory. Total time taken to complete TMT-B (capped at 180 s) was used as a measure of speeded mental flexibility, in the context of visual, attention and motor demands. Word generativity was measured using the total correct words generated, and response initiation and suppression was measured by the overall scaled score of the Hayling. The overall scaled score was selected because it integrates performance across response initiation, response suppression, and error components thereby capturing executive control more broadly.

Given the task demands of the Hayling, which requires semantic knowledge for meaningful completion, SD patients were not administered this task.

### Statistical analyses

Analyses were conducted using IBM SPSS Statistics, version 29.0 (SPSS Inc., Chicago, Ill., USA). Normality of variables was assessed using Shapiro-Wilks tests and visual inspection of histograms. Demographic variables (age, education) were analysed via one-way Analysis of Variance (ANOVA), with Games-Howell post-hoc testing to control for multiple comparisons, given unequal group sample sizes. Categorical measures (e.g., sex) were analysed using chi-squared tests. Group differences on disease severity, general cognition, language and executive function, were all compared using Kruskal–Wallis tests. Dunn’s procedure was applied to correct for multiple comparisons.

Given the non-normal distribution of SYDBAT scores, the frequency of impairment on each SYDBAT subtest was determined by identifying the cut off score for each subtest that signalled performance below the fifth percentile of the control-group distribution. The number and proportion of participants in each patient group meeting this criterion were then calculated.

To permit comparison of relative performance across SYDBAT subtests with different control distributions, raw scores were standardised using the control-group mean and standard deviation for each subtest. The resulting z scores indicated each participant’s performance relative to controls and were compared across subtests using paired Wilcoxon signed-rank tests. Given the non-normality of some score distributions, these analyses were interpreted as comparisons of relative standardised performance rather than as precise estimates of normally distributed impairment severity. Comparisons were made between naming and comprehension, naming and semantic association, naming and repetition, comprehension and semantic association, comprehension and repetition, and semantic association and repetition.

The relative and independent statistical contribution of language and executive functioning to SYDBAT performance in bvFTD was then examined using standard multiple regression. This method was chosen given its robustness in the presence of non-normal data [37]. Assumptions relating to independence, homoscedasticity, and multicollinearity were all met. For each model, ACE-III Semantic was entered as the measure of semantic functioning, while DSB, FAS, TMT-B, and Hayling Overall were entered as executive-function measures. All variables were entered simultaneously using the Enter method. Analyses were conducted separately for the possible and probable bvFTD groups. R-squared and beta coefficients were reported as indices of effect size.

## Results

### Demographics and clinical characteristics

The three patient groups were matched for age (all *p* values = 1.00; See Table A.1), years of education (F(3,298) = 1.88; *p* =0.134) and disease duration (e.g., years since symptom onset; *H*(2) = 4.95; *p* = 0.084). While the SD and control groups had a relatively equal distribution of males and females, the bvFTD groups showed clear male dominance (see Table 1). Disease severity (FRS), ranged from very mild to severe, with most of the patients in the moderate range. Disease severity in both bvFTD groups was greater than in the SD group (mean difference with probable bvFTD= − 1.77, 95% CI [− 2.42, − 1.13], *p* < 0.001; mean difference with possible bvFTD = − 1.40, 95% CI [− 2.14, − 0.67], *p* <0.001), but no significant difference was found between the two bvFTD groups (mean difference probable vs possible bvFTD = − 0.368, 95% CI [− 0.89, − 0.15], *p* = 0.212). On cognitive measure of disease severity, the reverse was found, where the SD group performed significantly below both bvFTD groups (total ACE-III, both *p* values ≤ 0.001; see Table A.2). Here, possible bvFTD outperformed probable bvFTD (*z* = − 3.22; *p* = 0.008).

**Table 1:** Demographic and clinical characteristics in possible bvFTD and probable bvFTD, SD and healthy controls

	Possible bvFTD (*n* = 43)	Probable bvFTD (*n* = 106)	SD (*n* = 42)	Controls (*n* = 112)	Group effect (*F* or *H* or χ2)	*p* value	Post hoc test results
Sex (m:f)	36:7	68:38	24:18	55:57	16.56^a^	<0.001	Male dominance in bvFTD
Age (years)	62.20 (8.65)	62.56 (8.07)	64.58 (7.07)	66.40 (6.89)	5.79^b^	<0.001	Both bvFTD < Controls
Education (years)	12.61 (3.35)	12.47 (3.09)	12.48 (2.90)	13.33 (2.69)	1.88^b^	0.134	Nil
Disease duration (years)	5.74 (3.60)	4.45 (2.98)	4.06 (2.07)	–	4.950^c^	0.084	SD < Possible bvFTD
FRS	0.02 (1.13)	− 0.35 (1.11)	1.42 (1.51)	–	29.87^b^	< 0.001	Both bvFTD < SD
ACE-III Total (/100)	85.19 (9.85)	77.61 (10.43)	61.38 (12.55)	94.87 (3.11)	201.19^c^	< 0.001	SD< Probable bvFTD < Possible bvFTD; All patient groups < Controls

Values are mean ± standard deviation. *ACE-III* Addenbrooke’s Cognitive Examination – Third Edition, *bvFTD* behavioural-variant frontotemporal dementia, *FRS* Frontotemporal Dementia Rating Scale, *SD* semantic dementia.

FRS severity ranges: Very mild (≥ 4.12); Mild (4.11 to 1.93); Moderate (1.91 to − 0.39); Severe (− 0.41 to − 2.57); Very severe (− 2.59 to − 4.98); Profound (≤ − 4.99). Number of missing values: 6 possible bvFTD, 3 probable bvFTD, 4 SD.

^a^: Chi-square test; ^b^: one-way ANOVA test;^c^: Kruskal–Wallis test.

### Cognitive profile

Patient groups performed significantly below healthy controls across all measures, with the exception of the ACE-III Visuospatial subscale and the ratio of TrailsB/TrailsA, where only the probable bvFTD group was reduced (ACE-III Visuospatial: *z* = − 6.03, *p* <0.001; ratio TrailsB/TrailsA: *z* =2.92; *p* =0.021; see Table 2 and Appendix A.2 and Appendix A.3). Among the patient groups, no significant differences were present in attentional capacity (all *p* values ≥ 0.22) or processing speed (all *p* values ≥ 0.372; see Appendix A.3). Some differences arose between groups in language, memory and executive function. The probable bvFTD group showed poorer working memory (DSB; *p* = 0.004) and slower performance on TMT-B (*p* = 0.009) than the SD group. The SD group was more impaired on the ACE-III language and memory subscales than both bvFTD groups (both *p* values < 0.001; Appendix A.2), and more impaired than the possible bvFTD group on ACE-III Fluency (*p* < 0.001; Appendix A.2).

**Table 2:** General cognitive and executive functioning tests in possible and probable bvFTD, SD and healthy controls

	Possible bvFTD (*n* = 43)	Probable bvFTD (*n* = 106)	SD (*n* = 42)	Controls (*n* = 112)	Group effect Kruskal–Wallis	*p*-values	Post hoc test (Bonferroni corrected)
ACE-III							
Attention (/18)	17.00 (2.00)	16.00 (2.00)	15.40 (1.85)	17.40 (0.40)	74.71	< 0.001	All patient groups < Controls
Memory (/26)	22.00 (6.50)	19.00 (6.00)	12.00 (6.75)	25.00 (2.25)	158.71	< 0.001	SD < Probable bvFTD < Possible bvFTD < Controls
Fluency (/14)	10.00 (6.50)	7.00 (5.00)	5.00 (5.00)	13.00 (1.25)	162.17	< 0.001	SD, Probable bvFTD < Possible bvFTD < Controls
Language (/26)	25.00 (2.00)	23.00 (4.00)	12.50 (5.85)	25.90 (1.00)	152.89	< 0.001	SD< both bvFTD < Controls
Visuospatial (/16)	15.00 (2.00)	15.00 (2.00)	16.00 (1.00)	16.00 (1.00)	38.33	< 0.001	Probable bvFTD < Controls
Digit span							
Forward (max span)	6.00 (2.00)	6.00 (2.00)	6.50 (2.75)	7.00 (2.00)	43.95	< 0.001	All patient groups < Controls
Backward (max span)	4.00 (2.00)	4.00 (2.00)	5.00 (1.00)	5.00 (2.00)	78.23	< 0.001	Probable bvFTD < SD; All patient groups < Controls
Trail Making Test							
Part A (sec)	39.00 (17.00)	45.50 (34.75)	39.00 (22.00)	30.00 (10.00)	63.78	< 0.001	All patient groups < Controls
Part B (sec)	95.00 (104)	154.00 (90.25)	85.50 (90.25)	72.00 (33.00)	88.12	< 0.001	Probable bvFTD < SD; All patient groups < Controls
Ratio (TrailsB/TrailsA)	2.47 (1.11)	2.65 (1.34)	2.54 (1.26)	2.26 (0.77)	9.14	0.027	Probable bvFTD < Controls
Verbal fluency							
Letter fluency (F,A,S)	31.00 (23.5)	22.00 (19.00)	25.00 (14.00)	48.00 (17.00)	129.58	< 0.001	Probable bvFTD < Possible bvFTD < Controls
Hayling							
Overall scaled score	5.00 (3.00)	3.00 (4.00)	–	6.00 (1.00)	93.86	< 0.001	Probable bvFTD < Possible bvFTD < Controls

Values are medians and interquartile ranges, given the non-normal distributions. Maximum scores of tests are indicated in parentheses where applicable. Hayling scaled score ranges: Poor: 3, Low average: 4, Moderate average: 5, Average: 6.

Number of missing values: Digit Span Backwards: 1 probable bvFTD; Trail Making Test: 1 Control; FAS letter fluency: 5 probable bvFTD; 5 SD; Hayling overall scaled score: 12 probable bvFTD, 1 possible bvFTD.

Both bvFTD groups were comparable to each other on the attention, language and visuospatial subscales of the ACE-III (all *p* values >0.196; Appendix A.2). On verbal fluency and memory subscales of the ACE-III, and on other measures of verbal generativity (FAS;* p* = 0.008; Hayling, *p* =0.033), the possible bvFTD group outperformed the probable bvFTD group (all *p* values <0.041).

### Language performance within bvFTD

**Fig. 1:**
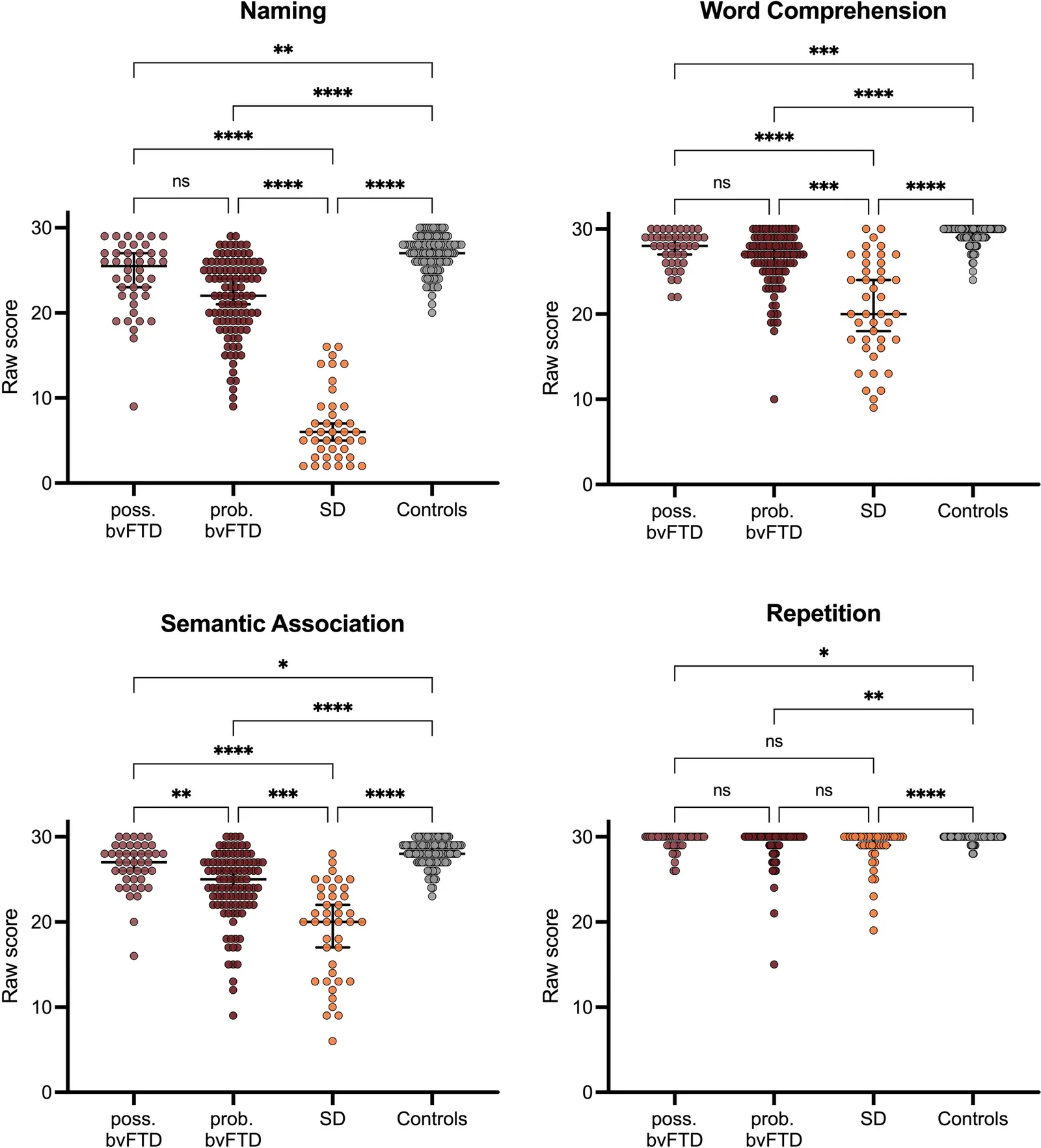
SYDBAT language performance across groups (possible bvFTD, probable bvFTD, SD patients and healthy controls); *P* values are denoted as follows: * = < 0.05; ** = < 0.01; *** = < 0.001; **** = < 0.0001

Group differences on the SYDBAT tasks are shown in Fig. 1. In line with our first hypothesis, probable bvFTD demonstrated significantly greater difficulties than possible bvFTD; however, this difference was only clearly observed on the semantic association subtest (*Z* = − 3.43,* p* = 0.004). While a trend towards a significant difference was found on naming (Z = − 2.61 *p* = 0.055), similar levels of deficit arose between the two bvFTD groups on comprehension (*Z* = − 2.15,* p* = 0.188), and repetition (*Z* = 0.130,* p* = 1.000).

Impairment frequencies varied across subtests and between bvFTD groups. In the possible bvFTD group, naming and comprehension deficits were observed in 28% and 26% of cases respectively), with around a fifth of patients showing impairments in semantic association (21%). The proportion of cases showing impaired word repetition was lower, at 14%. In the probable bvFTD group, approximately half of the patients showed impairments in naming (51%) and semantic association (49%). Deficits in comprehension were found in 41%. Again, the frequency of impairment for word repetition was lower, affecting 18% of patients. See Appendix A.6 for more detail.

### Language performance in SD and bvFTD

As expected, the SD group performed significantly lower than both the possible and probable bvFTD groups on the naming, comprehension and semantic association subtests (all *p* values < 0.001; see Fig. 1 and Appendix A.4). No differences were observed among the patient groups for the repetition subtest.

Within the SD group, naming performance was significantly more impaired than their performance on the comprehension and semantic association subtests (both *p* values ≤ 0.009; see Appendix A.7). Contrary to prediction, this pattern was not found in either of the two bvFTD groups. While naming, comprehension, and semantic association performance (all *p* values < 0.001) were significantly lower than repetition in the probable bvFTD group, no difference in performance was present between naming and the other subtests (naming vs comprehension: *p* =0.839; naming vs semantic association: *p* =0.354). For possible bvFTD, no differences were observed among any of the subtest pairs (all *p* values ≥ 0.131; see Appendix A.6), indicating a similar level of performance across all four subtests for this group.

### Semantic and executive associations with language deficits in bvFTD

To investigate the factors associated with language deficits in the two bvFTD groups, standard multiple regression analyses were conducted to assess the relative contributions of language (ACE-III Semantic) and executive functioning measures (DSB, FAS, TMT-B, Hayling Overall) to performance on each SYDBAT subtest. In the probable bvFTD group, significant models were obtained for SYDBAT naming (*F* (5, 84) = 7.42, *p* < 0.001; *R*^*2*^ = 31%), comprehension (*F* (5, 84) = 4.98, *p* < 0.001; *R*^*2*^= 23%), and semantic association tasks (*F* (5, 84) = 6.83, *p* < 0.001; *R*^*2*^ = 29%). In each case, ACE-III Semantic emerged as a significant predictor of subtest performance. The only additional predictor that approached significance was the Hayling test when completing the semantic association task (see Table 3). No significant linear relationship was found between performance on the SYDBAT repetition task and any of the predictor variables (*F* (5, 84) = 1.19, *p* = 0.320, *R*^*2*^ = 0.07).

**Table 3:** Significant predictors of language performance for probable bvFTD and possible bvFTD

Group	SYDBAT subscale	Variable	*B*	*SE B*	*β*	*p*	Sr^2^
Probable bvFTD	Naming	ACE-III Semantic	0.47	0.13	0.33	0.001*	0.10
	Comprehension	ACE-III Semantic	0.33	0.10	0.32	0.002*	0.10
	Semantic Association	ACE-III Semantic	0.44	0.12	0.34	0.001*	0.11
		Hayling Overall	0.41	0.21	0.21	0.056	0.03
Possible bvFTD	Naming	ACE-III Semantic	1.84	0.29	0.86	< 0.001*	0.39
	Comprehension	Hayling Overall	0.48	0.20	0.43	0.021*	0.09
		ACE-III Semantic	0.38	0.19	0.35	0.054	0.06
	Semantic Association	ACE-III Semantic	0.65	0.26	0.45	0.016*	0.11
	Repetition	ACE-III Semantic	0.37	0.10	0.69	< 0.001*	0.25
		Digits Backwards	0.38	0.12	0.55	0.004*	0.16
		Hayling Overall	− 0.26	0.10	− 0.48	0.012*	0.12

* Significant results indicated in bold; results combined across regression analyses

For possible bvFTD, significant results were observed for all four SYDBAT tasks: naming (*F* (5, 36) = 13.21, *p* < 0.001; *R*^*2*^ = 65%), comprehension (*F* (5, 36) = 5.31, *p* = <0.001; *R*^*2*^ = 42%), semantic association (*F* (5, 36) = 4.85, *p* = 0.002; *R*^*2*^=40%) and repetition (*F* (5, 35) = 5.06, *p* =0.001; *R*^*2*^ = 42%). Here, the ACE-III Semantic variable predicted language scores for naming, repetition, and semantic association. Unlike in the probable bvFTD patients, only performance on the Hayling task was a significant predictor for the Comprehension subtest, with semantic performance producing a marginally significant result (*p* = 0.054; see Table 3).

For the repetition subtest in possible bvFTD, effect sizes were constrained by restricted variability, given individual performances clustered near ceiling. Performance was predicted by the ACE-III Semantic variable and two measures of executive function: working memory (DSB), which provided a positive contribution, and response generation and inhibition (Hayling Overall), which showed a significant negative standardized coefficient (*β* = −0.48, *p* = 0.012). Given a negligible zero-order correlation between the Hayling and repetition performance (*r* = 0.11), this result suggested a suppression effect rather than a direct inverse association (i.e., indicating that performance on the Hayling may have accounted for variance in other measures unrelated to repetition performance). Collinearity diagnostics indicated acceptable tolerance and variance inflation factors (all VIFs < 2.0), suggesting that the observed suppression effect was not attributable to multicollinearity.

## Discussion

This study examined language performance in both possible and probable bvFTD using a unified battery of single word processing, with two primary aims: first, to determine if language changes observed in bvFTD resemble the pattern found in SD; and second, to examine the relative contributions of executive and semantic measures to single-word processing in bvFTD. Overall, results identified reductions in language performance in both bvFTD groups. Naming deficits were present in approximately half of individuals with probable bvFTD, with semantic association deficits also frequently observed in this group. In possible bvFTD, naming, comprehension and semantic association deficits were found in roughly a quarter of participants. Importantly, the pattern of relative impairment across subtests differed between bvFTD and SD. In the SD group, naming was significantly more impaired than the other subtests, whereas this pattern was not observed in either bvFTD group. Although semantic functioning was associated with single-word language performance in both bvFTD groups, some executive functioning measures also made an independent contribution.

Consistent with previous studies of probable bvFTD [5, 6, 38], group-level impairments were found across naming, single word comprehension, and semantic association. In contrast to earlier work, which only found significant reductions in naming, as compared with healthy controls, for cases with abnormal brain imaging (i.e., probable bvFTD) [11], comparable impairments were evident in the possible bvFTD group for the majority of language subtests. This earlier study, however, only included nine cases of “MRI-normal” bvFTD participants and only examined naming performance, rather than testing a broader range of single-word processing abilities. As noted within their study, intragroup variability was high, and while MRI-abnormal cases were clearly below healthy control performance, no significant differences were found when comparing directly between MRI abnormal and MRI normal patients [11]. While Kipps et al. [11] did not report individual frequencies of impairment, the current study found over a quarter of possible bvFTD patients showed naming and comprehension deficits. This suggests that language impairments may be present in both possible and probable bvFTD, although more commonly found in probable bvFTD. Given that the present analyses focused on baseline assessments (i.e., performance at a patient’s first assessment), these findings support the view that deteriorations in language can emerge early in the disease course rather than only at more advanced stages [21, 22].

An unexpected finding from the current study was the significant reduction in word repetition when comparing bvFTD groups with healthy controls. Although this finding differs from previous studies in bvFTD [15], reduced performance occurred in the context of very few repetition errors made by healthy controls on this task. Despite this, it is interesting to note that performances were substantially reduced for three participants with probable bvFTD and three participants with SD, but no participants with possible bvFTD. On review of audio files of these individuals, clear language difficulties were observed in the bvFTD cases (e.g., phonemic errors). This finding aligns with large cohort studies and genetic bvFTD series that have also reported a small but non-trivial subset of patients demonstrating impaired word and/or sentence repetition [39–41].

In line with previous research [5, 6], the probable and possible bvFTD groups showed significantly less impairment than the SD group on all subtests other than repetition, where the patient groups did not differ significantly. Contrary to prediction, however, the pattern of performance across the subtests typically seen within an individual with SD was not replicated in the two bvFTD groups. Only patients with SD showed a pattern wherein naming was significantly more impaired relative to the other subtests. Although the probable bvFTD group did show a similar preservation of repetition when compared to the other language tasks, patients with possible bvFTD demonstrated a flat profile of performance across tasks. This suggests that, while some overlap in language deficits may exist between SD and bvFTD, the nature of the language impairments may differ. Regarding the cognitive factors associated with the language declines, our results were consistent with past suggestions that both semantic and executive functioning deficits play a role in language performance [6]. In both bvFTD groups, single-word language performance was strongly associated with our measure of semantic abilities from the ACE-III and, to a lesser extent, with executive functioning measures. However, the models were more strongly predictive in possible bvFTD, explaining up to 65% of the variance, than in probable bvFTD, where they explained 23–31%. This could suggest that in the presence of significant frontotemporal brain changes (as required in a probable vs possible diagnosis), language performance may be influenced by a broader range of factors not captured by the current models. Supporting this interpretation, visuospatial performance was reduced in probable but not possible bvFTD on the ACE-III. If this result reflects true visuospatial decline rather than executive inefficiency, it may provide an additional explanatory factor to performance on subtests which require visual discriminations and picture matching (as in the comprehension and semantic association tasks of the SYDBAT). Predictors of word repetition differed between probable and possible bvFTD subgroups. While none of the variables considered were associated with performance in the probable bvFTD group, three contributions to the score were found in the possible bvFTD group. The strongest predictor, explaining the most unique variance, was semantic knowledge (from the ACE-III), followed by working memory capacity. This finding is consistent with the reliance of repetition on intact lexical–semantic representations and short-term maintenance. In contrast, a negative contribution was found from the Hayling, which taken together with the negligible direct association with repetition performance and limited variance within this sample, suggests that individual differences in response initiative and suppression may modulate the contribution of other cognitive processes.

The relatively high frequency of semantic association impairment in probable bvFTD, together with evidence of significant associations with both semantic and executive measures is interesting to note. One possibility is that a subset of individuals with bvFTD has genuine disruption of semantic knowledge, potentially reflecting involvement of anterior temporal systems. Alternatively, poor performance may arise from executive difficulties affecting the controlled retrieval, selection, and monitoring of semantic information, rather than loss of the underlying representations themselves, or a combination of these two factors. Although the regression findings indicate that both semantic and executive measures were associated with this aspect of word-processing performance, it is important to note that the semantic and executive measures included do not constitute process-pure measures, instead sharing to some extent verbal, retrieval, selection, attentional, and monitoring demands. Verbatim responses and qualitative error data were not retained, preventing analysis of perseverative, off-task, semantic, phonological, or other error types. Given the overlap in task demands, it is therefore not possible to definitively distinguish degradation of semantic knowledge from impaired executive control of semantic processing. Future studies, combining qualitative analysis of error types together with measures designed to contrast automatic and controlled semantic processing, may aid in resolving this question.

### Clinical Implications and Future Directions

The occurrence of single-word language impairments in both the probable and possible bvFTD groups suggests that current diagnostic criteria for bvFTD may benefit from revision. Incorporating assessments of naming and broader semantic knowledge into diagnostic evaluations may improve diagnostic confidence and provide insights into the likely clinical course.

Appreciating that language deficits may arise from a combination of executive and linguistic aspects may inform more effective intervention strategies. Declines in naming and semantic abilities can lead to everyday communication problems, such as word-finding difficulties and semantic paraphasias [42]. Interventions targeting language in bvFTD may need to consider not only linguistic elements but also executive demands (e.g., providing bvFTD patients with structured, cued response options) and support initiation and organisation of information to help address potential difficulties with coherency and goal-directed expression.

Future research will benefit from adopting fine-grained approaches, such as examining error types, to further distinguish semantic and executive contributions (e.g., perseverative responding from various types of semantic errors). In the only study to do this to-date, patients with probable bvFTD were found to produce high rates of associative errors and omissions, similar to SD patients [6]. Expanding this to examine the type of errors made by those with possible bvFTD may provide further opportunities to distinguish between bvFTD types and identify which individuals are likely to progress from possible to probable bvFTD. Longitudinal studies are needed to characterise trajectories of language decline and identify early predictors of rapid progression. Finally, expanding assessment beyond single-word processing to include connected speech and narrative language will strengthen links to everyday communication function.

## Conclusions

Language performance is significantly impacted in both possible and probable bvFTD groups and should not be overlooked in the clinical examination when a diagnosis of bvFTD is considered. Although bvFTD and SD share broad similarities in language performance, the disproportionally severe naming impairment characteristic of SD is not observed in bvFTD. Language deficits in bvFTD appear primarily semantically driven, with executive functioning exerting a modulating influence. Together, these findings highlight the importance of considering both language and executive processes when characterising cognitive change in bvFTD.

## Data Availability

The conditions of the original ethics approval signed by participants do not permit public archiving of data supporting the conclusions of this study. Readers seeking access to the data should contact the FRONTIER group directly. Access can only be granted via a formal data sharing agreement to named individuals in accordance with ethical procedures governing the reuse of sensitive data.

## Appendix A

See Table A.1, A.2, A.3, A.4, A.5, A.6 and A.7

**Table A.1:** Follow-up independent samples t-tests of probable and possible bvFTD, SD, and healthy controls for Age

Group comparisons	Age
Probable bvFTD vs Possible bvFTD	*t*(3) = 0.26, *p* = 1.00
Probable bvFTD vs SD	*t*(3) = − 1.46, *p* = 1.00
Possible bvFTD vs SD	*t*(3) = 1.45, *p* = 1.00
Probable bvFTD vs Controls	*t*(3) = − 3.80, ***p***** = 0.002***
Possible bvFTD vs Controls	*t*(3) = − 3.13, ***p***** = 0.019***
SD vs Controls	*t*(3) = 1.36, *p* = 1.00

*** Significant values indicated in bold

**Table A.2:** Follow-up Dunn’s tests with Bonferroni corrections for probable and possible bvFTD, SD groups and healthy controls for ACE-III Total and ACE-III Subscales

Comparisons	Total	Attention	Memory	Fluency	Language	Visuospatial
Probable bvFTD vs Possible bvFTD	***Z*** ** = − 3.22,** *** p*** ** = 0.008***	*Z* = − 2.11,* p* = 0.213	***Z*** ** = − 2.71,** *** p*** ** = 0.041***	***Z*** ** = − 3.59,** *** p*** ** =0.002**	*Z* = − 1.85,* p* = 0.386	*Z* = − 2.14,* p* = 0.196
Probable bvFTD vs SD	***Z*** ** = 3.89,** *** p*** ** =0.001***	*Z* = 0.49,* p* = 1.000	***Z*** ** = 4.03,** *** p*** ** < 0.001***	*Z* = 1.92,* p* = 0.055	***Z*** ** = 6.50,** *** p*** ** < 0.001***	***Z*** ** = − 3.53,** *** p*** ** =0.002***
Possible bvFTD vs SD	***Z*** ** = 5.95,** *** p*** ** < 0.001***	*Z* = 2.33,* p* = 0.118	***Z*** ** = 5.64,** *** p*** ** < 0.001***	***Z*** ** = 4.60,** *** p*** ** < 0.001**	***Z*** ** = 7.01,** *** p*** ** < 0.001***	*Z* = − 1.19,* p* = 1.000
Probable bvFTD vs Controls	***Z*** **= − 11.09,** ***p*** **< 0.001***	***Z*** ** = − 7.69,** *** p*** ** < 0.001**	***Z*** ** = − 9.49,** *** p*** ** < 0.001***	***Z*** ** = − 10.90,** *** p*** ** < 0.001**	***Z*** ** = − 7.22,** *** p*** ** < 0.001***	***Z*** ** = − 6.03,** *** p*** ** < 0.001***
Possible bvFTD vs Controls	***Z*** ** = − 5.10,** *** p*** ** < 0.001***	***Z*** ** = − 3.69,** *** p*** ** =0.001**	***Z*** ** = − 4.44,** *** p*** ** < 0.001***	***Z*** ** = − 4.62,** *** p*** ** < 0.001**	***Z*** ** = − 3.59,** *** p*** ** < 0.001***	*Z* = − 2.40,* p* = 0.097
SD vs Controls	***Z*** **= − 12.21,** ***p*** **< 0.001***	***Z*** ** = − 6.46,** *** p*** ** < 0.001**	***Z*** ** = − 11.17,** *** p*** ** < 0.001***	***Z*** ** = − 10.10,** *** p*** ** < 0.001**	***Z*** **= − 11.96,** ***p*** **< 0.001***	*Z* = − 0.96,* p* = 1.000

*** Significant values indicated in bold

**Table A.3:** Follow-up Dunn’s tests with Bonferroni corrections for probable and possible bvFTD, SD groups and healthy controls for the executive functioning and other cognitive measures

Comparisons	Attentional Capacity (DSF)	Working memory (DSB)	Processing Speed (TMT-A)	Mental flexibility (TMT-B)	TMT Ratio (TrailsB/TrailsA)	Verbal fluency (FAS)	Response initiation and inhibition (Hayling overall scaled score)
Probable bvFTD vs Possible bvFTD	*Z* = 0.94,* p* = 0.314	*Z* = − 2.28,* p* = 0.023	*Z* = 1.87,* p* = 0.372	*Z* = 2.07,* p* = 0.230	*Z* = *0.3*9, *p* = 1.00	***Z*** ** = − 3.20,** *** p*** ** = 0.008***	*Z* = − 2.54,* p* = 0.033
Probable bvFTD vs SD	*Z* = − 0.2.09,* p* = 0.220	***Z*** ** = − 3.40,** *** p*** ** < 0.001***	*Z* = 1.86,* p* = 0.376	***Z*** ** = 3.16,** *** p*** ** = 0.009***	*Z* =*0.8*4*, p*= 1.00	*Z* = − 0.67,* p* = 1.000	*–*
Possible bvFTD vs SD	*Z* = − 0.14,* p* = 1.000	*Z* = − 0.96,* p* = 0.339	*Z* = 0.01,* p* = 1.000	*Z* = − 0.93,* p* = 1.000	*Z* = *0.3*8*, p* = 1.00	*Z* = 2.03,* p* = 0.256	**–**
Probable bvFTD vs Controls	***Z*** ** = − 0.6.59,** *** p*** ** < 0.001***	***Z*** ** = − 8.81,** *** p*** ** < 0.001***	***Z*** ** = 7.82,** *** p*** ** < 0.001***	***Z*** ** = 9.25,** *** p*** ** < 0.001***	***Z*** ** = 2.92** ***, p*** ** = 0.021**	***Z*** ** = − 10.77,** *** p*** ** < 0.001***	***Z*** ** = − 9.57,** *** p*** ** < 0.001***
Possible bvFTD vs Controls	***Z*** ** = − 3.02,** *** p*** ** = 0.015***	***Z*** ** = − 4.35,** *** p*** ** < 0.001***	***Z*** ** = 4.04,** *** p*** ** < 0.001***	***Z*** ** = − 4.91,** *** p*** ** = 0.001***	*Z* = 1.81*, p* = 0.423	***Z*** ** = − 4.99,** *** p*** ** < 0.001***	***Z*** ** = − 4.79,** *** p*** ** < 0.001***
SD vs Controls	***Z*** ** = − 0.2.83,** *** p*** ** = 0.028***	***Z*** ** = − 3.16,** *** p*** ** = 0.002***	***Z*** ** = 3.99,** *** p*** ** < 0.001**	***Z*** ** = 3.75,** *** p*** ** = 0.001***	*Z* = 1.34*, p* = 1.00	***Z*** ** = − 7.12,** *** p*** ** < 0.001***	**–** ^a^

*** Significant values indicated in bold. ^a^: The SD group did not complete the Hayling test

**Table A.4:** SYDBAT test scores in possible bvFTD and probable bvFTD, SD and healthy controls

	Possible bvFTD (*n* = 43)	Probable bvFTD (*n* = 106)	SD (*n* = 42)	Controls (*n* = 112)	Group effect	*p*-value	Post hoc tests among groups (Bonferroni corrected)
Naming (/30)	25.50 (4.00) *Z* = − 1.29	22.00 (6.00) *Z* = − 2.44	6.00 (5.00) *Z* = − 9.45	27.00 (2.00)	169.63	< 0.001	SD < bvFTD; All patient groups < Control
Comprehension (/30)	28.00 (2.00) *Z* = − 1.33	27.00 (4.00) *Z* = 2.54	20.00 (8.75) *Z* = − 7.44	30.00 (1.50)	129.68	< 0.001	SD <bvFTD; All patient groups < Control
Semantic Association (/30)	27.00 (4.00) *Z* = − 1.02	25.00 (5.00) *Z* = − 2.71	20.00 (9.00) *Z* = − 6.12	28.00 (2.00)	133.51	< 0.001	Probable bvFTD < Possible bvFTD; SD < bvFTD; All patient groups < Control
Repetition (/30)	30.00 (1.00) *Z* = − 1.14	30.00 (1.00) *Z* = − 1.80	30.00 (1.00) *Z* = − 3.23	30.00 (0.00)	29.68	< 0.001	All patient groups < Control

Values are median and interquartile range. Maximum raw scores of the SYDBAT subscales are indicated in parentheses. All *p*-values were obtained from Kruskal–Wallis tests on raw scores. When “bvFTD” is used, it refers to both possible and probable bvFTD. For z score calculation, controls means and standard deviations are as follows: Naming = 27.05 (2.06); Comprehension =29.27 (1.12); Repetition = 29.87 (0.41); Semantic Association = 28.14 (1.50)

SYDBAT: Sydney Language Battery Test. Number of missing values: SYDBAT naming: 1 SD; SYDBAT repetition: 1 probable bvFTD; SYDBAT comprehension: 1 probable bvFTD; SYDBAT semantic association: 2 probable bvFTD, 1 SD

**Table A.5:** Follow-up Dunn’s tests with Bonferroni corrections for probable and possible bvFTD, SD groups and healthy controls for subscales of the SYDBAT tests

Comparisons	Naming	Repetition	Comprehension	Semantic association
Probable bvFTD vs Possible bvFTD	*Z* = − 2.61, *p* = 0.055	*Z* = 0.130, *p* = 1.000	*Z* = − 2.15, *p* = 0.188	***Z*** ** = − 3.43, ** ***p*** ** =0.004***
Probable bvFTD vs SD	***Z*** ** = 6.30, ** ***p*** ** < 0.001***	*Z* = 2.13, *p* = 0.201	***Z*** ** = 4.17, ** ***p*** ** < 0.001***	***Z*** ** = 4.07, ** ***p*** ** < 0.001***
Possible bvFTD vs SD	***Z*** ** = − 7.47, ** ***p*** ** < 0.001***	*Z* = 1.67, *p* = 0.570	***Z*** ** = 5.30, ** ***p*** ** < 0.001***	***Z*** ** = 6.29, ** ***p*** ** < 0.001***
Probable bvFTD vs Controls	***Z*** ** = − 8.11, ** ***p*** ** < 0.001***	***Z*** ** = − 3.71, ** ***p*** ** = 0.001***	***Z*** ** = − 8.19, ** ***p*** ** < 0.001***	***Z*** ** = − 8.33, ** ***p*** ** < 0.001***
Possible bvFTD vs Controls	***Z*** ** = − 3.50, ** ***p*** ** = 0.003***	***Z*** ** = − 2.92, ** ***p*** ** = 0.021***	***Z*** ** = − 4.02, ** ***p*** ** < 0.001***	***Z*** ** = − 2.86, ** ***p*** ** = 0.025***
SD vs Controls	***Z*** ** = − 12.38, ** ***p*** ** < 0.001***	***Z*** ** = − 4.93, ** ***p*** ** < 0.001***	***Z*** ** = − 10.35, ** ***p*** ** < 0.001***	***Z*** ** = − 10.33, ** ***p*** ** < 0.001***

*** Significant values indicated in bold

**Table A.6:** Frequency of impairment across SYDBAT subtests for all patient groups

Patient group	Naming	Repetition	Comprehension	Semantic association	Impaired on at least 3 subtests
Probable bvFTD	54/106 (51%)	19/105 (18%)	43/105 (41%)	51/104 (49%)	34/104 (33%)
Possible bvFTD	12/43 (28%)	6/42 (14%)	11/43 (26%)	9/43 (21%)	8/43 (19%)
SD	41/41 (100%)	10/42 (24%)	33/42 (79%)	34/41 (83%)	31/42 (74%)

Impairment is defined as scoring below 5th percentile from healthy control data

**Table A.7:** Pairwise comparisons between SYDBAT subtests for possible and probable and SD groups using Wilcoxon rank sum tests

Comparisons between SYDBAT subtests	Possible bvFTD	Probable bvFTD	SD
Naming vs Comprehension	*Z* = 0.02; *p* = 0.981	*Z* = 0.20; *p* = 0.839	***Z*** ** = 2.61; ** ***p*** ** = 0.008**
Naming vs Semantic	*Z* = 1.51; *p* = 0.131	*Z* = − 0.93; *p* = 0.354	***Z*** ** = 4.62; ** ***p*** ** < 0.001**
Naming vs Repetition	*Z* = 0.53; *p* = 0.595	***Z*** ** = 3.72; ** ***p*** ** < 0.001**	***Z*** ** = 4.96; ** ***p*** ** < 0.001**
Comprehension vs Semantic	*Z* = 1.50; *p* = 0.134	*Z* = − 1.63; *p* = 0.104	*Z* = 1.85; *p* = 0.065
Comprehension vs Repetition	*Z* = 0.77; *p* = 0.441	**Z = 3.49; ** ***p*** ** < 0.001**	***Z*** ** = 3.90; ** ***p*** ** < 0.001**
Semantic vs Repetition	*Z* = − 0.11; *p* = 0.915	**Z = − 3.71; ** ***p*** ** < 0.001**	***Z*** ** = − 3.66; ** ***p*** ** < 0.001**

Significant results indicated in bold
